# Hatching plasticity is associated with a more advanced stage at hatching in an *Ambystoma* with terrestrial eggs

**DOI:** 10.1002/ece3.11160

**Published:** 2024-03-18

**Authors:** Kimberly D. Treadaway, Rebecca E. Hale

**Affiliations:** ^1^ Biology Department University of North Carolina Asheville Asheville North Carolina USA

**Keywords:** amphibian, embryo, extended development, hatching plasticity, larva

## Abstract

Hatching plasticity allows animals to initiate hatching in response to environmental cues including predation, flooding, and hypoxia. In species with terrestrial eggs but aquatic larvae, hatching plasticity often manifests as extended development of embryos when water is not available. Although these effects are taxonomically widespread, little attention has focused on differences in plasticity across closely related species with terrestrial and aquatic embryos. We propose that the terrestrial embryonic environment favors slower and prolonged development and, consequently, that we should see differences in development between closely related species that differ in where they lay their eggs. We test this hypothesis by comparing embryonic development between two mole salamanders, *Ambystoma opacum* and *A. annulatum*. Most *Ambystoma* lay eggs submerged in ponds but *A. opacum* lays its eggs on land, where hatching is triggered when eggs are submerged by rising pond levels. Embryos of both species were reared under common laboratory conditions simulating both aquatic and terrestrial nest sites. Consistent with our hypothesis, we found that *A. opacum* embryos exhibited slower development and took longer to hatch than *A. annulatum* embryos in both rearing environments. Furthermore, we observed in *A. opacum* a plasticity in hatching stage that was absent in *A. annulatum*. Our results indicate that the terrestrial‐laying *A. opacum* has evolved slower and prolonged development relative to its aquatic‐laying congener and suggest that embryonic survival in the unpredictable terrestrial environment may be facilitated by developmental plasticity.

## Graphical abstract

Hatching plasticity and the ability to extend development until suitable hatching conditions are available is widespread among amphibians with terrestrial eggs. However, whether this plasticity is unique to species with terrestrial eggs has not been explored thoroughly. We compared embryonic development of two Ambystomatid salamanders across two environments and observed slower and extended development as well as greater plasticity in the species with terrestrial eggs, suggesting the terrestrial environment may favor unique developmental traits.

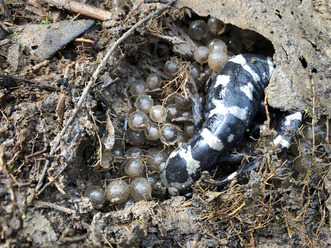

## 1 INTRODUCTION

Hatching plasticity is widespread among both invertebrate and vertebrate taxa and allows developing embryos to initiate hatching in response to their environment (Warkentin, 2011a). Across diverse species, physical stimulation (DiMichele & Taylor, 1981; Griem & Martin, 2000; Oyarzun & Strathmann, 2011; Warkentin, 2005), chemical signals such as predator and conspecific cues (Miner et al., 2010; Poo & Bickford, 2014; Smith & Fortune, 2009; Warkentin, 1995), and salinity (Allen et al., 2017; Haramura, 2016) can trigger early hatching. Embryos that can initiate hatching in response to environmental cues may face tradeoffs; while early hatching allows embryos to escape predation, hatchlings may be smaller or less developed (Delia et al., 2019; Poo et al., 2023), which can be associated with greater larval predation risk and lower larval survival (Delia et al., 2019; Gomez‐Mestra et al., 2008; McCoy et al., 2011; but see Vonesh & Bolker, 2005).

In species with terrestrial eggs, an important environmental cue triggering hatching is flooding (Gomez‐Mestra et al., 2008; Warkentin, 2011b). Many fish and amphibians lay eggs out of water on emergent vegetation, in seasonally dry pond beds, or above the water line (Martin & Carter, 2013; Warkentin, 2011a). These embryos experience higher oxygen availability while not having to contend with aquatic predators. However, they also risk desiccation and predation by terrestrial predators (Delia et al., 2019; Poo & Bickford, 2014). Furthermore, inundation must be carefully matched with embryonic development, as embryos submerged prematurely may die (Poo et al., 2023; Pyburn, 1970), but delayed inundation may result in desiccation or starvation (Marco & Blaustein, 1998; Touchon et al., 2010).

For some amphibians, terrestrial egg‐laying allows adults to deposit eggs early, in pond beds before they fill with seasonal rains. This early laying gives their embryos a head start on development over larval competitors whose eggs must be submerged. In addition, breeding need not be coordinated with pond flooding, the timing of which may vary widely from year to year. However, terrestrial egg‐laying may be risky if ponds fill late, or if they fill and then subsequently dry (Marco & Blaustein, 1998). Embryos with the ability to extend or stall development may have an advantage: if the pond fills soon after fertilization, the embryos could develop as would an aquatic species, whereas if the pond fills late, the embryos could delay hatching and even continue to grow. Indeed, extended development has been documented in a wide range of species with terrestrial eggs (reviewed in Martin, 1999). For example, embryos of the two‐toed amphiuma (*Amphiuma means*; Gunzburger, 2003), marbled salamander (*Ambystoma opacum*; Hale et al., 2016), and the California grunion (*Leuresthes tenuis*; Moravek & Martin, 2011) continue to develop if they are not submerged. However, ultimately all these species require water upon hatching and the longer the air exposure, the fewer may survive (e.g., *Ambystoma gracile*, Marco, 2001).

Although hatching plasticity and extended development have been described in diverse taxa, in amphibians, such research has primarily focused on species with terrestrial eggs. Whether species with terrestrial embryos exhibit different patterns of embryonic development than their relatives with aquatic embryos has been largely ignored. However, we might expect embryos laid on land to have evolved different patterns of development or sensitivities to their environments than aquatic embryos, and evidence from Ambystomatid salamanders suggests such a divergence. Marbled salamander (*Ambystoma opacum*), a species with terrestrial embryos, exhibit a much wider range of developmental stages and ages at hatching than the aquatic egg‐laying spotted salamander (*A. maculatum*), when reared under conditions mimicking both aquatic and terrestrial habitats (Hale et al., 2016).

These observations suggest that terrestrial and aquatic embryos have different levels of plasticity in response to the environment. Furthermore, if flooding of terrestrial nests typically happens after embryos have reached a viable stage for hatching, then the terrestrial environment may favor embryos that develop more slowly, as longer embryonic development and/or slower development may result in greater yolk conversion efficiency and subsequently larger larval size (Van Leeuwen et al., 2017).

**FIGURE 1:**
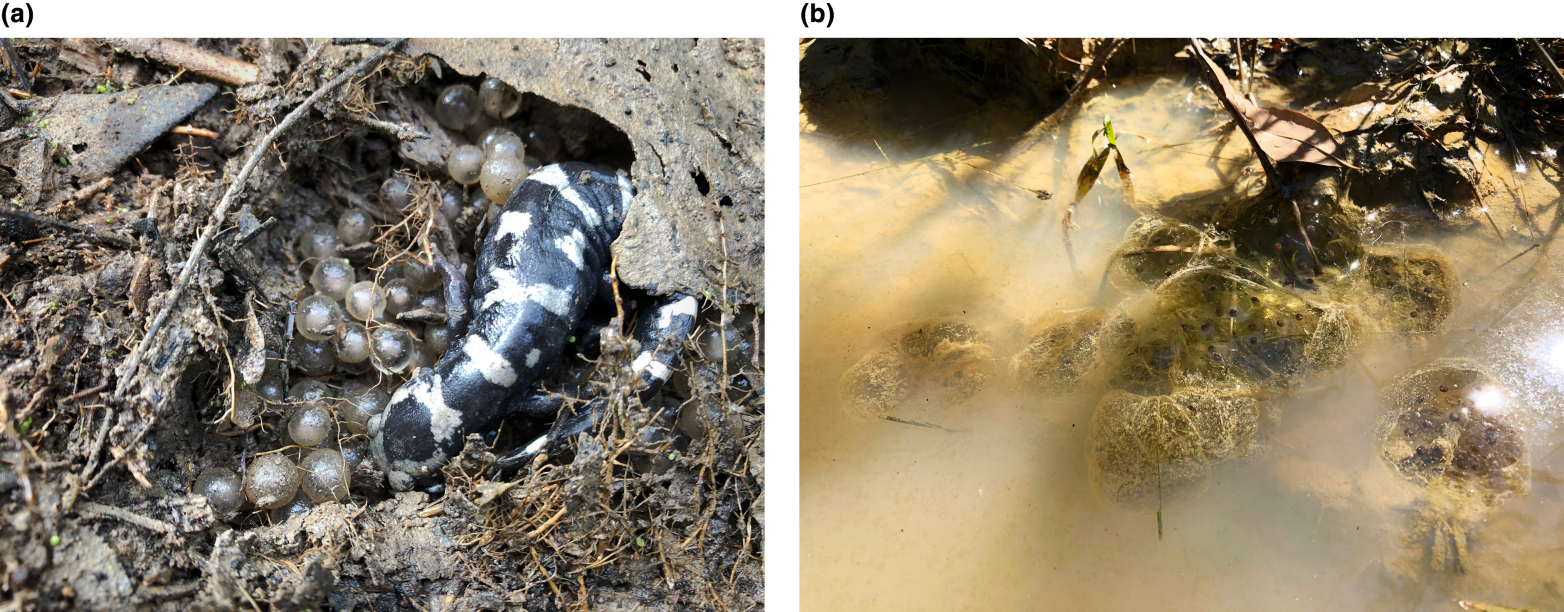
(a) Female marbled salamander (*Ambystoma opacum*) with embryos. Nest was under a log on the margins of a wildlife pond in Ozark‐St. Francis National Forest, Pope County, AR. (b) Ringed salamander (*A. annulatum*) egg masses attached to sticks in a wildlife pond in Ozark‐St. Francis National Forest, Franklin County, AR. Photos by R. E. Hale.

Salamanders of the genus *Ambystoma* are well‐suited to test the hypothesis that terrestrial egg laying is associated with greater hatching plasticity, slower development, and the ability to extend development. All *Ambystoma* have internal fertilization and aquatic larvae and, thus, are restricted to breeding in or near water. Two lineages, marbled salamander and the two flatwoods salamanders (*A. cingulatum* and *A. bishopi*) lay eggs terrestrially, without jelly, in excavated nests under logs (Figure 1a) or at the base of dense vegetation (Anderson & Williamson, 1976; Petranka & Petranka, 1981). Marbled and flatwoods salamanders migrate to wetland depressions before they fill with fall and winter rains. As rains flood the wetland, nests are submerged and embryos hatch (Petranka et al., 1982). However, most species breed in ephemeral ponds and lay their fertilized eggs on submerged vegetation surrounded by a thick layer of jelly that protects them from predation and desiccation (Figure 1b; Marco & Blaustein, 1998; Takahashi & Ruszala, 2023). The ringed salamander (*A. annulatum*) lays its eggs in water, but does so in the fall like the terrestrially‐laying species.

Environmentally sensitive hatching has been demonstrated in numerous *Ambystoma*. Both of the terrestrial‐laying lineages exhibit delayed hatching when exposed to air for prolonged periods (Anderson & Williamson, 1976). However, evidence for delayed hatching among aquatic‐laying species is mixed. Hale et al. (2016) found that spotted salamander (*A. maculatum*) embryos do not delay hatching when reared in air. In contrast, Marco and Blaustein (1998) showed that embryos of the northwestern salamander (*A. gracile*), which lays its eggs submerged, can delay hatching if exposed to air upon short term pond drying, but they do not extend their development while doing so.

The purpose of the current study is to test the hypothesis that species with terrestrial eggs exhibit greater plasticity in embryonic development and hatching, including a greater tendency to prolong development. We compared embryonic development of the fall‐breeding, terrestrial laying marbled salamander (*A. opacum*) with the fall‐breeding, aquatic‐laying ringed salamander (*A. annulatum*) under conditions simulating both aquatic and terrestrial environments. Greater plasticity, slower development, or prolonged development in the marbled salamander compared to the ringed salamander would support the hypothesis that the terrestrial environment affects developmental traits.

## 2 METHODS

### 2.1 Study species and collection

Ringed salamander (*A. annulatum*) embryos were collected from multiple sites in Arkansas in September 2019 under permits issued by Ozark National Forest (Permit # OZF‐FW‐FY19‐06) and the Arkansas Game and Fish Commission (Permit # 06182191). Following a wet summer, September was considerably hotter and drier than typical in northern and eastern Arkansas (National Weather Service, 2023). Following the first light rain in approximately 20 days, spawning was observed the night of September 25 in Ozark National Forest (Franklin County site). Previous inspection of nearby sites had observed no spermatophores or egg masses prior to this night, suggesting spawning did not occur in this region prior to this date. In total, 32 ringed salamander masses were collected for the experiment. Seven masses were collected from the Franklin County site and eight from another Ozark National Forest site in Washington County on September 26. An additional 12 masses were collected from McIlroy Madison Wildlife Management Area (Madison County) on September 27, and 5 clutches from one site in Baxter County on September 28. The 15 masses collected from Ozark National Forest were shipped overnight to the University of North Carolina Asheville on September 26, were assigned September 26 as their first day of development, and were identified as Harrison stages 9 and 10 (Harrison, 1969) on September 27. The remaining 16 masses were driven to North Carolina, were assigned September 27 as their first day of development, and were placed in experimental treatments on September 30. Embryos from all sites appeared of similar, pre‐gastrulation stage as though fertilization occurred at all sites on the same night. Indeed, no spermatophores or masses were found prior to September 25 and no adults were observed after that date.

**FIGURE 2:**
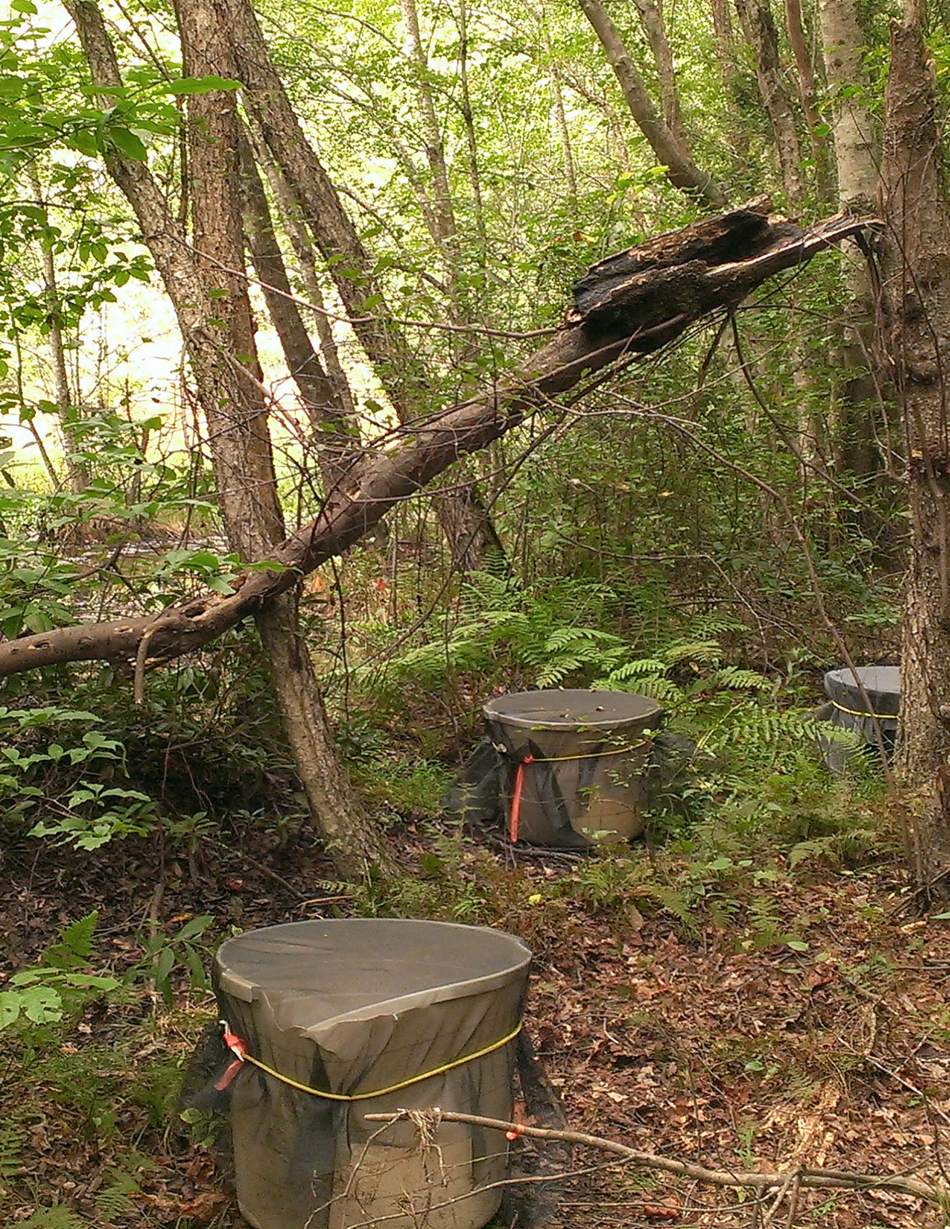
Oviposition bins like those used for marbled salamander (*Ambystoma opacum*) at the Pulaski County (Little Rock) site in 2019. Bins were 0.6 m diameter × 0.5 m height and contained 10–15 cm depth of leaf litter, had holes in the bottom to prevent water retention, and were covered with netting attached by an elastic cord. One or two gravid females and one to two males were placed inside, under leaf letter. We returned 3–7 days after placement to inspect for deposited eggs. Bins in photograph were used in North Carolina in 2014 by R.E. Hale.

Marbled salamander (*A. opacum*) embryos were collected from similar sites in Arkansas between October 14 and 19, 2019 under the same permits; however, only six nests were located. One clutch was collected from St. Francis National Forest (Phillips County) on October 14, four clutches from the City of Little Rock (Pulaski County) on October 15 and 19, and one clutch from Ozark National Forest (Pope County) on October 19. At the Little Rock site, gravid females but no nests were observed on October 13. This same day, some gravid females were placed in 0.6 m diameter × 0.5 m height plastic enclosures for oviposition (Figure 2). Enclosures contained 10–15 cm depth of leaf litter, had holes in the bottom to prevent water retention, and were covered with netting attached by an elastic cord. Two days later, one nest was found in an enclosure and two additional nests were found under logs. More adults were subsequently placed in enclosures on October 17 and a fourth clutch was collected from an enclosure on the 19th. All clutches were driven to UNC Asheville on October 20 and clutches were assigned a first day of development corresponding to their day of collection.

Diel temperature ranges were obtained from National Weather Service weather stations near each collection site (National Weather Service, 2023). During the collection of ringed salamander embryos, the average air temperature ranged from a low of 15°C to a high of 25°C near the four collection sites. During the collection of marbled salamander embryos, the average air temperatures ranged from a low of 4°C to a high of 24°C near the three sites.

### 2.2 Embryo manipulation and measurement

All embryos were collected as either an egg mass, constituting a group of presumed half or full siblings surrounded by a jelly layer (ringed salamander), or a clutch of half or full siblings found together in a nest site (marbled salamander). Hereafter, we refer to both of these grouping as clutches. Clutches were kept separate in gas‐permeable bags for transport back to Asheville, NC. On the campus of the University of North Carolina Asheville, all embryos were counted and live embryos were selected to reach our final sample sizes for ringed salamander (*n* = 1240 embryos from 31 clutches) and marbled salamander (*n* = 240 from 6 clutches). The unequal sample sizes of the two species resulted from their breeding occurring a month apart, and our inability to collect a comparable number of marbled salamander clutches during our second collection trip after we had already set up replicates of ringed salamander.

Removal of jelly from ringed salamander embryos was necessary to ensure the embryos of both species were reared with similar exposure to the treatment environment. This was accomplished by gently pulling the jelly apart and releasing intact, encapsulated embryos into tap water (aged to allow chlorine to evaporate before use). Marbled salamander embryos are not encased in jelly so were not separated, but were rinsed in the same tap water to remove sediment. While we recognize that the manipulation experienced by embryos differed between species, embryos of both species were handled, either for jelly or sediment removal, before being placed in treatments.

Each clutch was divided into two treatment groups of 20 embryos. One group was placed in a 50‐mL specimen jar submerged in ~40 mL aged tap water (water treatment), whereas the other group was placed in an identical jar with ~1 mL aged tap water to prevent desiccation (air treatment). These two treatments were identical to the “saturated” and “air” treatments in Hale et al. (2016). In the water treatment, air was pumped into the jar via an airstone inserted through the jar lid, submerged in the water, and connected through tubing to an aquarium pump. The low but constant air flow ensured the water was oxygen saturated. Identical lids with airstones were placed on jars in the air treatment. However, due to difficulty maintaining air pressure from our pumps when connected to a high number of jars, airstones on air treatment jars were not connected to an air pump and lids were left loose on the jars to allow air circulation. All jars were randomly situated in a Conviron CMP6050 growth chamber (Controlled Environments Limited, Manitoba) at 20°C, 80% relatively humidity, with a 12 h:12 h light: dark cycle. The temperature reflects the approximate median daily temperature from both collection periods. In the air treatment, embryos were moistened, as needed, to prevent desiccation; embryos rested in a small puddle of water in the bottom of the jar, but were not submerged.

We inspected all embryos under a dissecting microscope daily and recorded the number of live, dead, and hatched embryos. Embryos were scored as dead if the egg membranes were opaque or if the yolk and embryo were collapsed and non‐spherical. The latter often occurred in early stage embryos. The minimum and maximum embryonic stage (Harrison, 1969) of live, developing embryos were recorded for each jar and were later converted to median stage to assign the jar a single stage value. Later stage embryos (i.e., >Harrison stage 20) sometimes appeared stunted and were scored as dead after multiple days of no apparent developmental progress. This method of identifying dead embryos may bias estimates of longevity; however, we assume it biases all species/treatments equally and, furthermore, latency to death was not a metric we analyzed. More saliently, this method may reduce the median stage on a given day when stunted embryos were included in staging; however, we do not expect such an underestimate of median stage to differ among species/treatment combinations.

The embryonic stage upon hatching was recorded for each individual and the first five hatchlings per jar were euthanized in MS‐222 and preserved in Shandon™ Glyo‐Fixx™ (Richard Allan Scientific, Kalamazoo, MI, USA) before being vacuum dried for 48 h and weighed to the nearest 10μg on a Mettler‐Toledo XP2U microbalance (Mettler‐Toledo LLC, Columbus, OH, USA).

### 2.3 Analyses

Survivorship of embryos of the two species placed in the air and water treatments was analyzed in a generalized linear model with binomial error that included species, treatment, and species x treatment interaction as independent variables. Among those embryos that hatched, we evaluated age, stage, and mass at hatching for differences between site and/or collection date (hereafter referred to as “site”), and did so separately for each species. We used a generalized linear mixed effect model with site and treatment as fixed effects and embryo within clutch as a random effect. Inclusion of the random effect allowed us to use data collected from individual hatchlings while accounting for embryos being collected from the same clutch and, thus, sharing at least one parent. We specified Gaussian error distribution for stage and mass and Poisson error distribution for age. Within the ringed salamander, we tested for differences between embryos collected from Franklin and Washington Counties within Ozark National Forest, McIlroy Madison WMA, and Baxter County. Within the marbled salamander, we tested for differences between embryos collected from Phillips County within St. Francis National Forest, Pope County within Ozark National Forest, and two dates on which we collected from the site in Pulaski County (October 15 and October 19).

Treatment and species effects on embryonic stage, age, and mass at hatching were evaluated using generalized linear mixed effect models. Models included species, treatment, and species x treatment interaction as fixed effects. Clutch and site identity were included as a random effects. We specified Gaussian error distribution for stage and mass and Poisson error distribution for age. For these analyses, only embryos that survived to hatching were included.

We also evaluated the data for evidence of tradeoffs between stage and mass at hatching by examining whether the relationship between these two variables differed between species or treatment. We evaluated the species separately and treated mass as the dependent variable in a generalized linear mixed effect model stage, treatment, and their interaction as independent variables and embryo within clutch as a random effect.

Statistical models were evaluated in R using the lm and glm functions of the stats package (R Core Team, 2022) and the glmer and lmer functions of the lme4 package (Bates et al., 2015). Effects of independent variables were evaluated by model comparison with likelihood ratio tests using the anova function in the stats package. The likelihood ratio test statistic is approximately *χ* ^2^ distributed with 1 degree of freedom; therefore, we report *χ* ^2^ values (Agresti, 1996). Marginal means of the response variables were estimated using the emmeans package of R (Lenth, 2020).

## 3 RESULTS

### 3.1 Hatching success

**FIGURE 3:**
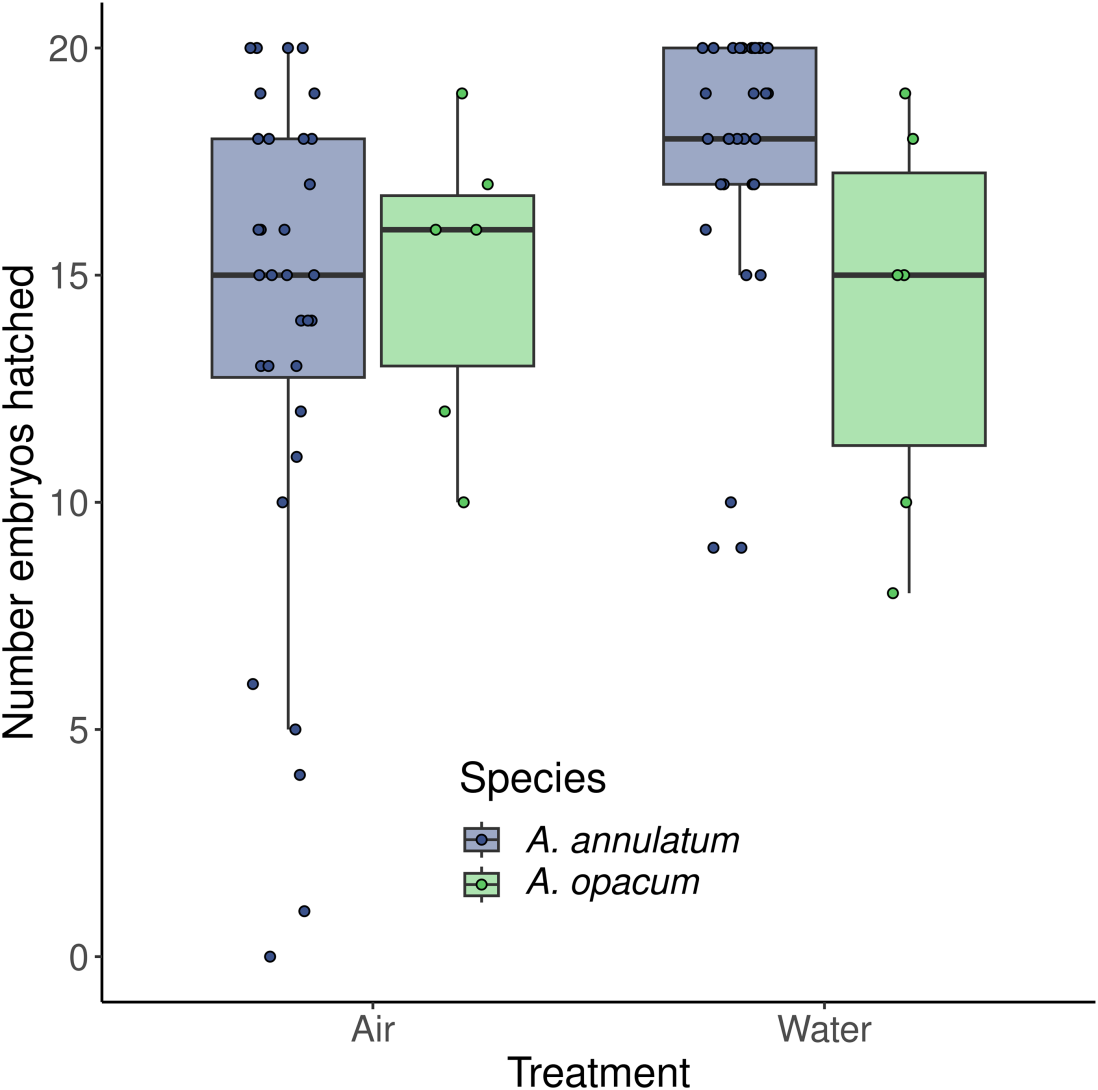
Number of embryos per jar, out of 20, that hatched in air and water rearing environments. Horizontal spread is added to increase visibility of individuals points. The air treatment simulates the terrestrial environment in which *Ambystoma opacum* typically deposits embryos. The water treatment simulates the aquatic environment in which *A. annulatum* typically deposits embryos. There were no significant differences in number that hatched between species or treatments.

A total of 1280 ringed salamander embryos (64 jars) and 240 marbled salamander embryos (12 jars) were evaluated for survival to hatching. Survivorship to hatching differed significantly between air and water treatments ($\chi_{1}^{2}$ = 6.1, *p* = .01), but did not differ between species ($\chi_{1}^{2}$ = 0.44, *p* = .51). Survivorship was higher in water (mean **±** SD: 17.1 ± 3.5 embryos) than in air (14.1 ± 5.2 embryos; Figure 3). Although there was no statistical interaction ($\chi_{1}^{2}$ = 1.85, *p* = .17), the difference between treatments appears driven by the large treatment effect in *A. annulatum*, of which there were considerably more replicates.

### 3.2 Site, date, and collection effects

One ringed salamander mass from Ozark National Forest was excluded from all subsequent analyses because some embryos exhibited anomalously early hatching (Harrison stages 23 and 24) in the air treatment. Furthermore, stage and age at hatching were evaluated for the 972 ringed salamander and 175 marbled salamander embryos that survived to hatching. Mass at hatching was evaluated for five embryos from each jar, such that sample size for mass measurements was 310 ringed salamander embryos and 60 marbled salamander embryos.

**FIGURE 4:**
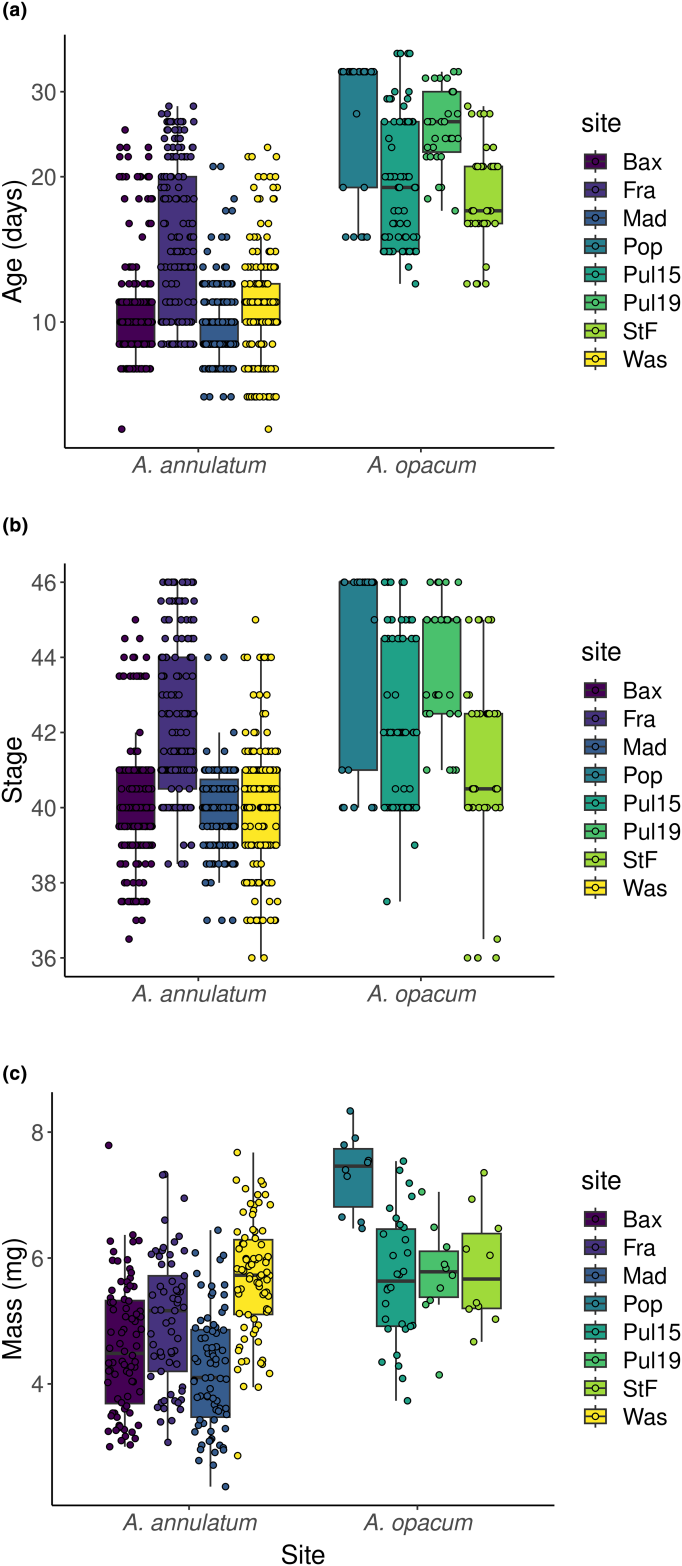
(a) Age, (b) Harrison (1969) stage, and (c) mass at hatching for embryos of ringed (*Ambystoma annulatum*) and marbled (*A. opacum*) from six sites in Arkansas. Ringed salamander embryos were collected from Baxter County (Bax), Franklin County (Fra), Madison County (Mad), and Washington County (Was). Marbled salamander embryos were collected from Pulaski County (Pul15 and Pul19), Pope County (Pop), and Phillips County (StF). The two boxplots for Pulaski County represent two days of collection. Embryos from air and water treatments are combined within a site.

In both species, when and from where embryos were collected influenced traits at hatching (Table 1, Figure 4). Ringed salamander embryos from Franklin County hatched 5 days older and one stage more developed, but with comparable mass to embryos from other sites. These Franklin County embryos were collected 1 to 3 days earlier than embryos from other sites. In contrast, marbled salamander embryos collected 4 and 5 days sooner—from Phillips and Pulaski Counties—took 4–5 days fewer to hatch and hatched 1–2 stages less advanced, but did not differ significantly in mass from embryos from the other collections.

**TABLE 1:** Results of likelihood ratio tests evaluating the effect of collection site on three hatchling variables: age, stage, and mass at hatching.

Dependent variable^a^	*χ* ^2^	*p*
*Ambystoma annulatum*		
Age	44.32	1.3 × 10^−9^
Stage	23.89	2.6 × 10^−5^
Mass	44.15	1.4 × 10^−9^
*Ambystoma opacum*		
Age	8.66	.034
Stage	8.18	.042
Mass	5.51	.14

*Note*: There were four collection sites for ringed salamanders (*A. annulatum*) and three for marbled salamander (*A. opacum*) species, but the two collection dates from one site (Pulaski County) were separated in these analyses. The likelihood ratio test statistic approximately *χ* ^2^ with one degree of freedom, so *χ* ^2^ and associated probabilities are reported here. Although treatment effects were included in these models, their importance is evaluated in more complete models, reported in Table 2.

^a^ Full model took the form *y* = site + treatment + (1|clutch).

We collected embryos on two dates and using two methods at the Pulaski County site; therefore, we performed t tests to evaluate whether collection date or collection method (from enclosures or from natural nest sites) affected our metrics within these four clutches. We found differences in age and stage, but not mass, between collection dates (Table 2). We also found differences in age, stage, and mass at hatching between embryos collected from natural nests and those from enclosures. Embryos collected from enclosures took longer to hatch, hatched at a more advanced stage, but with lower mass.

**TABLE 2:** Results of Welch two sample *t* tests to evaluate the effects of collection date and whether or not embryos were collected from females placed in enclosures.

Variable	Group mean		*t*	df	*p*
**Date of collection**	**Early**	**Late**			
Age (days)	19.78	25.65	−5.46	73.3	6.26 × 10^−7^
Stage^a^	41.83	43.89	−5.34	67.1	1.20 × 10^−6^
Mass (mg)	5.66	5.74	−0.26	20.1	.79
**Enclosure**	**Yes**	**No**			
Age	23.91	18.95	−4.44	99.7	2.36 × 10^−5^
Stage	41.56	43.23	−4.40	109.5	2.50 × 10^−5^
Mass	6.00	5.37	2.16	37.6	.037

*Note*: Tests evaluate embryos from four clutches collected at the Pulaski County site, with *N* = 160 for age and stage and *N* = 40 for mass.

^a^ Harrison (1969).

### 3.3 Stage, age, and mass at hatching

**FIGURE 5:**
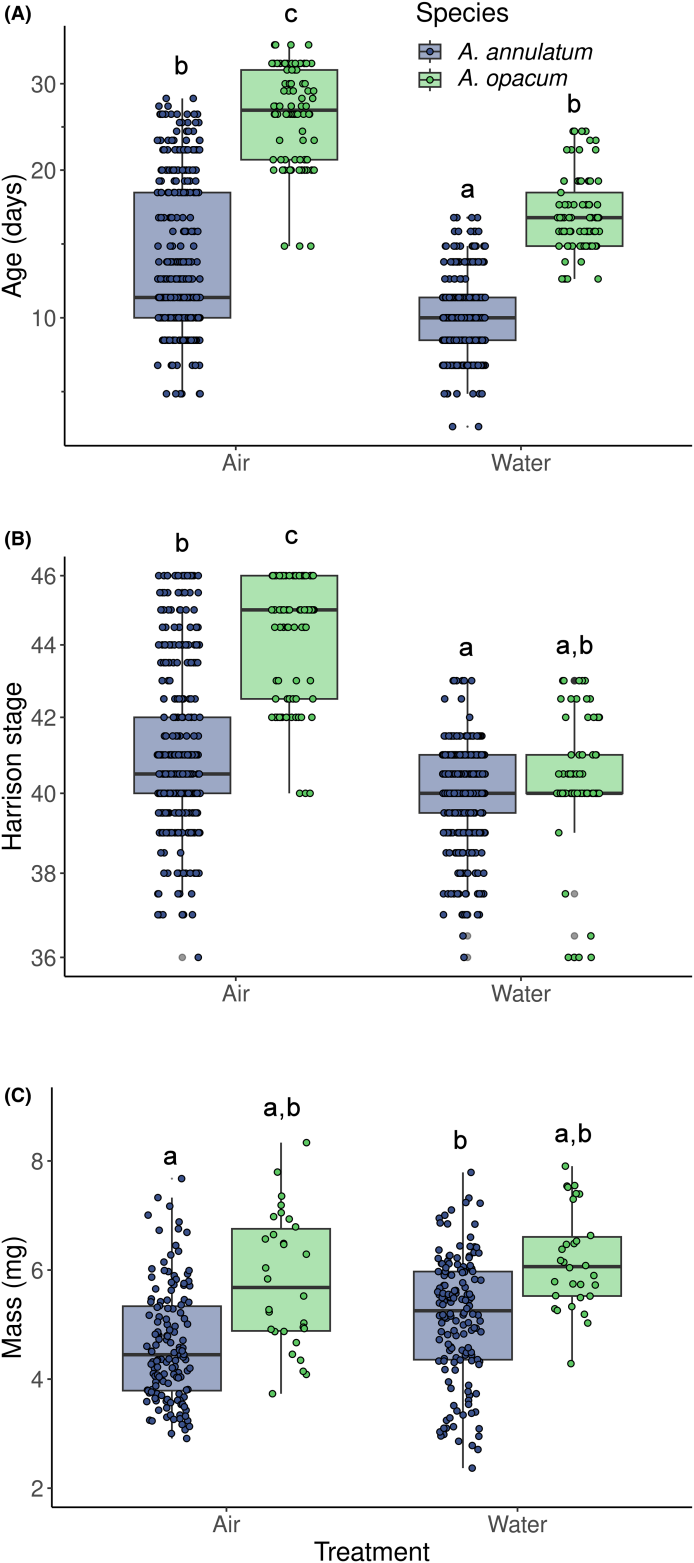
(A) Age, (B) Harrison (1969) stage, and (C) mass at hatching for embryos of ringed (*Ambystoma annulatum*) and marbled (*A. opacum*) salamanders reared in either moist air or water, at 15°C. The air treatment simulates the terrestrial environment in which *A. opacum* typically deposits embryos. The water treatment simulates the aquatic environment in which *A. annulatum* typically deposits embryos. Horizontal spread is added to increase visibility of individuals points. Boxplots with the same letter were not significantly different, as evaluated using the Tukey method and the eemeans function in R. See text for a summary of differences between treatments.

Age at hatching was affected by an interaction between species and treatment; treatment affected age at hatching in both species, but had a stronger effect in marbled salamander (Table 3). In air, marbled salamander hatched nearly 2 weeks later than ringed salamander (marginal mean age (95% CI): 27.4 days (23.1, 32.1) vs. 13.7 days (11.8, 15.8)). In water, this difference was reduced to about 1 week (17.1 days (14.6, 20.3) in marbled salamander vs. 10.1 days (8.6, 11.6) in ringed salamander; Figure 5A).

**TABLE 3:** Results of likelihood ratio tests evaluating species and treatment (air or water rearing environment) effects on three hatchling variables: age, stage, and mass at hatching.

Variable	*χ* ^2^	*p*
Age at hatching^a^		
Species	13.02	.0003
Treatment	439.79	<1.0 × 10^−15^
Species × treatment	15.30	9.17 × 10^−5^
Stage at hatching		
Species	5.40	.020
Treatment	231.70	<1.0 × 10^−15^
Species × treatment	165.03	<1.0 × 10^−15^
Mass at hatching		
Species	5.96	.015
Treatment	20.17	7.1 × 10^−6^
Species × treatment	0.076	.78

*Note*: The likelihood ratio test statistic approximates a *χ* ^2^ distribution with one degree of freedom, so *χ* ^2^ and associated probabilities are reported here.

^a^ Full model took the form *y* = species + treatment + species × treatment + (1|clutch) + (1|site).

Stage at hatching also was affected by an interaction between species and treatment, with a species difference in the air, but not water treatment. In air, marbled salamander hatched approximately 3.5 Harrison stages further developed than ringed salamander (marginal mean stage (95% CI): 44.5 (43.2, 45.8) vs. 41.1 (39.8, 42.4)), but there was no difference between species in stage at hatching when reared in water (40.7 (38.9, 41.5) vs. 40.2 (38.9, 41.5); Figure 5B).

Species and treatment both had significant effects on mass at hatching, but there was no species × treatment interaction. In both air and water, marbled salamander hatchlings had an average mass approximately 1 mg greater than ringed salamander and masses in both species were slightly greater in water than air (marginal mean mass (mg) (95% CI) air: 5.93 (5.06, 6.80) vs. 4.63 (3.79, 5.48); water: 6.31 (5.44, 7.19) vs. 5.09 (4.24, 5.94); Figure 5C).

**FIGURE 6:**
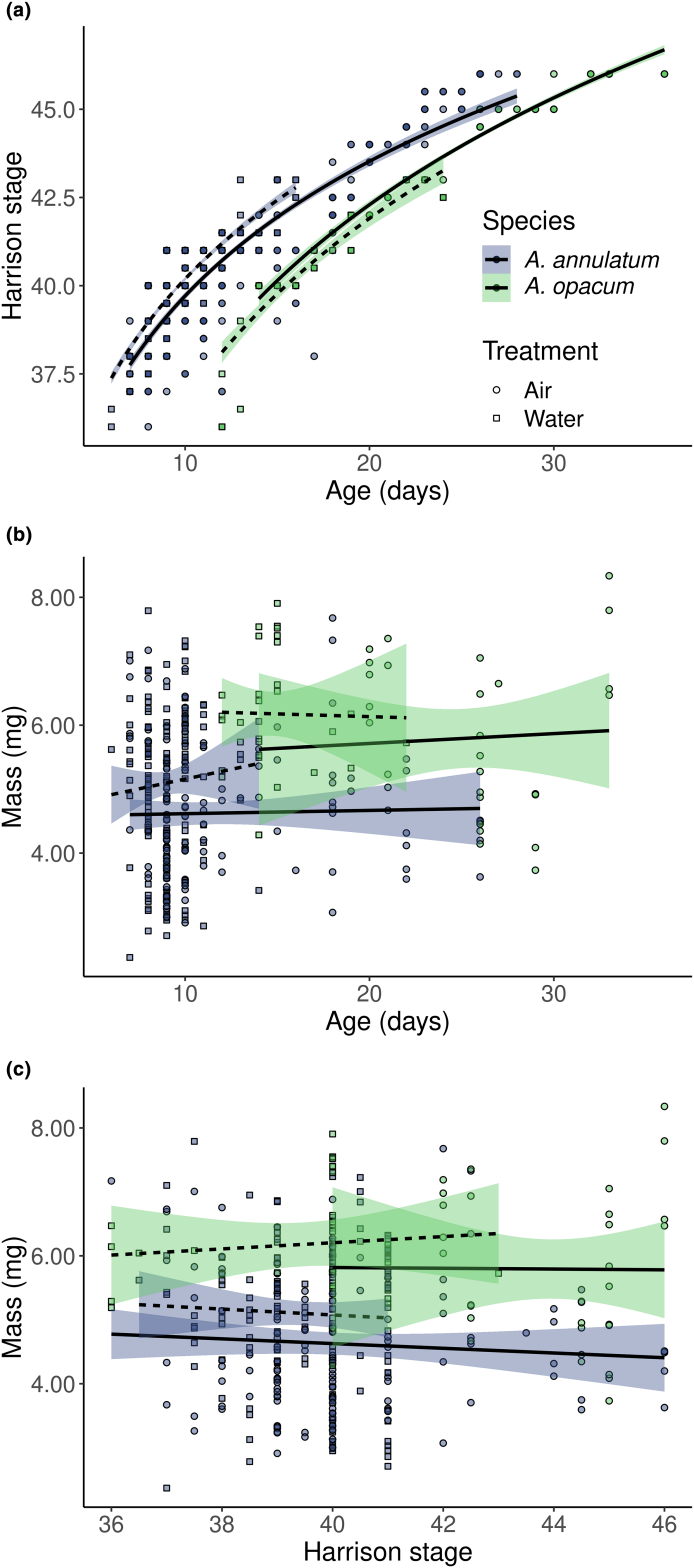
Relationships between (a) age and stage at hatching and (b) age and mass at hatching, and (c) stage and mass at hatching for each species and treatment combination. Dashed lines indicate regression for water treatment, solid lines for air treatment. Stage at hatching increased with log(age) at hatching in both species, with a greater positive slope for marbled than ringed salamanders. There was no relationship between stage and mass in marbled salamander (*Ambystoma opacum*). There was a weak negative relationship (slope = −0.09, effect size *η*2 = 0.05) between stage and mass for ringed salamander (*A. annulatum*). The relationship between age and mass at hatching are visualized but not statistically evaluated, as we decided a priori to test for relationships only between age and stage at hatching and between mass and stage at hatching.

In addition to differing in their response to air and water treatments, the two species differed in development rate. Marbled salamanders took longer than ringed salamanders to reach the same developmental stages (Figure 6a). Furthermore, the relationship between age and stage at hatching differed between the two species. In both species, once they reached the stage of first hatching (~37.5), embryos that took longer to hatch were able to reach more advanced developmental stages. However, marbled salamanders advanced development further for every day they continue to grow, as evident in the significant interaction between log(age) and treatment on stage at hatching (Table 4).

**TABLE 4:** Results of likelihood ratio tests evaluating the relationship between age and stage at hatching.

Variable	Estimate^a^	χ ^2^	*p*
Log(age)	4.94	1549.50	<1.0 × 10^−15^
Species (*Ambystoma opacum*)	−10.25	18.51	1.69 × 10^−5^
Treatment (water)	0.42	74.76	<1.0 × 10^−15^
Age × species	2.96	229.54	<1.0 × 10^−15^
Age × treatment		2.19	.14

*Note*: Stage was treated as the dependent variable while age, species, and treatment were treated as independent variables. Coefficients of model terms are provided for the independent variables in the best fitting model. The likelihood ratio test statistic approximates a *χ* ^2^ distribution with one degree of freedom, so *χ* ^2^ and associated probabilities are reported here.

^a^ Coefficients are provided for terms in the best fit model.

### 3.4 Tradeoffs between stage and mass at hatching

Examining the effects of treatment across the three hatchling measurements revealed an interesting pattern. In marbled salamander, embryos reared in air took longer to hatch and hatched at a more advanced stage, but at the apparent cost of body mass (Figure 5). However, examining the relationship between stage and mass at hatching more closely within each species, we found little evidence of a stage and mass tradeoff. In ringed salamander, there was a weak negative relationship between stage and mass at hatching (coefficient of stage on mass = 0.10 ± 0.04 SE, effect size *η* ^2^ = 0.05; Table 5, Figure 6c). This analysis also confirmed the treatment effect on mass, but only for ringed salamander, and its effect size was also quite small (*η* ^2^ = 0.04). There was no relationship between stage and mass at hatching in marbled salamander. The absence of stage or treatment effects on hatchling mass for marbled salamanders suggests these embryos, which extend development in air and hatch at a more advanced stage, do so without cost to body mass.

**TABLE 5:** Results of likelihood ratio tests evaluating the relationship between mass and stage at hatching, with treatment as a covariate.

Variable	Estimate^a^	*χ* ^2^	*p*
*Ambystoma annulatum*			
Stage	−0.038	6.11	.01
Treatment (water)	0.42	10.40	.001
Stage × treatment		0.066	.80
*A. opacum*			
Stage		0.25	.62
Treatment (water)		0.18	.67
Stage × treatment		0.40	.53

*Note*: Species were evaluated separately. Coefficients of model terms are provided for the independent variables in the best fitting model. The likelihood ratio test statistic approximates *a χ* ^2^ distribution with one degree of freedom, so *χ* ^2^ and associated probabilities are reported here.

^a^ Coefficients are provided for terms in the best fit model.

## 4 DISCUSSION

Embryos experience widely different conditions whether they are deposited terrestrially or aquatically. Aquatic embryos are situated in their larval habitat throughout development, whereas terrestrial embryos transition from air to water, which can occur well before or long after they are competent to hatch. Embryos that must wait for nest flooding may reach larger larval sizes if they slow their development and convert more of their yolk to body mass (Van Leeuwen et al., 2017). As a result, a variable timing of flooding may favor greater plasticity and both slower and extended development in species with terrestrial embryos relative to those with aquatic embryos.

Our results reveal greater developmental plasticity, slower development, and extended development in the terrestrial‐nesting marbled salamander, compared to its aquatic‐laying congener, the ringed salamander. Slowing and extending development regardless of environment may be advantageous for marbled salamander embryos, who may only rarely find themselves submerged during early stages of development, such as when they are laid relatively late in the season or when ponds flood early. Flooding of nests can occur weeks to months after oviposition (Hassinger et al., 1970; Jackson et al., 1989; Worthington, 1968); however, marbled salamander embryos reach a viable hatching stage in one to four weeks, depending on temperature (e.g., Kaplan & Crump, 1978). Therefore, terrestrial laying may mean embryos wait two to three times the period necessary for viability before hatching. Our data suggest that they use the remaining time, perhaps until yolk reserves are depleted, to reach more advanced developmental stages. However, when nests do flood early in development, their plasticity allows them to transition sooner to the larval stage.

Previous work by Hale et al. (2016) showed greater plasticity and slower development in marbled salamander compared to another aquatic‐laying, but winter breeding species, the spotted salamander. However, because the breeding season of these two species differ, they did not interpret the differences to be due to egg‐laying environment. Instead, they argued that the much earlier breeding by marbled salamanders could relax selection for fast development. Both ringed and marbled salamanders breed in the fall, yet the two aquatic‐laying species, ringed and spotted salamanders, hatched sooner and at earlier Harrison stages than marbled salamanders, contradictory to the seasonal explanation for the previous species difference. Instead, the combined results of this study and that of Hale et al. (2016) point to the terrestrial environment as the ecological factor associated with greater developmental plasticity and extended development.

At the proximate level, sensitivity of embryos to dissolved oxygen may explain why submerged embryos do not reach more advanced stages in marbled salamander than in ringed salamander. Hatching is triggered when embryos experience oxygen limitation (Petranka et al., 1982; Warkentin, 2011b) and oxygen limitation should be more acute both at more advanced developmental stages (due to larger body mass) and in water relative to air. Therefore, we expect terrestrial embryos to experience oxygen limitation later in their development. This mechanism may allow the embryos to respond differently in water than air, growing further when in the terrestrial environment.

Terrestrial nesting, whether it confers a competitive advantage to larvae or simply gives offspring an early start on growth, renders embryos vulnerable to wetlands that fill late or never fill. We have shown that these embryos have the capacity to continue to develop while they wait. Many amphibians with terrestrial eggs bypass the larval stage altogether and extended development may be an intermediate step in the evolution of such direct development. In the absence of water, it is conceivable that embryos could develop through metamorphosis, provided they have enough yolk (Callery et al., 2001). Indeed, although the developmental progression through embryonic stages varies across direct‐developing species (discussed in Callery et al., 2001), direct‐developing frogs and salamanders both progress through a tadpole (frog) or gilled larva (salamander) form before they hatch (Callery et al., 2001; Kerney, 2011; Marks & Collazo, 1998). In this way, extended development is, in fact, a characteristic of direct development. An analysis of hatching plasticity generally, and extended development specifically, across closely related species with and without direct development could provide insight into the stages of the evolution of direct development and how selection shapes them.

Furthermore, terrestrial eggs are associated with parental care in many amphibians and parental care may play an important role in embryonic development of *Ambystoma*. Marbled salamander females remain at nests after egg laying and abandon nests at flooding, if not earlier (Jackson et al., 1989; Petranka, 1990). Attendance by females reduces embryo mortality due to predation and fungal infection (Croshaw & Scott, 2005). Maternal care, coupled with the relatively long period between fertilization and hatching, could favor offspring that can prolong the embryonic stage. While marbled salamander is the sole species in the genus with parental care, it and the two flatwoods salamanders (*A. cingulatum* and *A. bishopi*) lay eggs terrestrially. Therefore, the influence of parental care, independent of the effect of the terrestrial environment, could be evaluated by comparing development across these species. Extended development has been demonstrated in *A. cingulatum* (Anderson & Williamson, 1976), but whether its embryos develop more slowly than its aquatic congeners is not known and could be a valuable next step in this research.

We have shown that hatching plasticity in marbled salamander allows its embryos to extend development on land and hatch at a more advanced stage than embryos reared in water. Furthermore, we have shown that marbled salamanders exhibit hatching plasticity absent in its congener, ringed salamanders. Our results, combined with those of Hale et al. (2016) and Marco and Blaustein (1998), suggest that this developmental plasticity, and indeed extending development to hatch at a more advanced stage, is associated with terrestrial egg‐laying in this genus. Future work comparing embryonic development among species with divergent embryo environments and, more generally, divergent reproductive behavior may identify suites of coevolving traits and possibly shed light on the evolution of direct development.

## AUTHOR CONTRIBUTIONS

**Kimberly D. Treadaway:** Conceptualization (equal); data curation (equal); formal analysis (equal); investigation (equal); methodology (equal); visualization (equal); writing – original draft (lead). **Rebecca E. Hale:** Conceptualization (equal); data curation (equal); formal analysis (equal); investigation (equal); project administration (lead); supervision (lead); writing – review and editing (lead).

## CONFLICT OF INTEREST STATEMENT

Neither author has a conflict of interest with respect to this project or in the publication of these data. Both authors contributed equally to designing and collecting data. REH analyzed the data and prepared figures and tables. KT prepared the first version of the manuscript for her undergraduate thesis, which was revised by REH for publication. This study was funded by a University of North Carolina System Interinstitutional Planning Grant to R. E. Hale and by the UNC Asheville Biology Department. The work was approved by the UNC Asheville Institutional Animal Care and Use Committee (Protocol 2019‐Res04). The authors have no competing interests to declare.

## Data Availability

The dataset generated during the current study is available from Dryad [http://doi.org/10.5061/dryad.f4qrfj72k] and the R script used to analyze the data is available from Zenodo [http://zenodo.org/records/10070149].
